# When the night is not long enough: soil drying constrains nighttime tree rehydration, setting a physical limit to stomatal regulation

**DOI:** 10.1093/treephys/tpag119

**Published:** 2026-08-20

**Authors:** Julian Schoch, Lorenz Walthert, Peter Lehmann, Andrea Carminati

**Affiliations:** Institute of Terrestrial Ecosystems, ETH Zurich, 8092 Zurich, Switzerland; Forest and Soil Ecology, Swiss Federal Institute for Forest, Snow and Landscape Research WSL, 8903 Birmensdorf, Switzerland; Institute of Terrestrial Ecosystems, ETH Zurich, 8092 Zurich, Switzerland; Institute of Terrestrial Ecosystems, ETH Zurich, 8092 Zurich, Switzerland

**Keywords:** dendrometer, leaf water potential, sap flow, transpiration, tree water use, vapour pressure deficit

## Abstract

Plants are often assumed to optimize carbon assimilation against water loss through stomatal regulation, responding instantaneously to varying atmospheric demand and soil water supply. However, a growing body of work highlights the importance of nighttime recovery processes for understanding plant water use, yet nighttime processes are not considered in existing supply–demand models. Here, we extend an existing supply–demand model by including flux imbalances between atmospheric demand and soil supply buffered by plant water storage. The flux imbalances accumulate during the day and are compensated overnight. Applying the conceptual model to field data from *Quercus pubescens Willd.*, we show that progressive soil drying constrains the rate of nighttime rehydration. Precisely, below a critical soil water potential threshold (ψ_soil,crit_), nighttime rehydration begins to decline because rehydration rates become too low relative to the duration of the night (‘the night is not long enough’). This threshold marks a transition beyond which maintaining high transpiration rates becomes increasingly risky. The observations show that stomatal regulation begins near this threshold, pointing to the concomitance between daytime stomatal regulation and limited nighttime rehydration. These results suggest that progressive soil drying constrains nighttime rehydration and reveal a critical hydraulic threshold that coincides with the onset of stomatal regulation and highlight that plant water use is constrained not only by daytime fluxes but also by nighttime recovery.

## Introduction

Climate change intensifies hot and dry conditions, increasing tree mortality (Hammond et al. 2022) and reshaping forest structure and carbon storage (McDowell and Allen 2015). Forests act as a major carbon sink (Pan et al. 2011) and, together with other terrestrial ecosystems, contribute substantially to evapotranspiration over land (Jasechko et al. 2013), thereby playing a critical role in regulating global carbon and water cycles. This regulation occurs to a great extent through stomata, which control the exchange of vapour and CO₂ between vegetation and the atmosphere. Rising vapour pressure deficit (VPD) and drying soils reduce vegetation productivity by inducing stomatal closure (Jung et al. 2010, Novick et al. 2016, Liu et al. 2020). However, understanding the mechanisms behind the physiological and environmental drivers of stomatal regulation, including the interaction between VPD and soil drying, remains challenging (Carminati and Javaux 2020, Grossiord et al. 2020). Specifically, there is a mismatch between: (i) studies predicting stomatal closure using the instantaneous water supply–demand framework, which focus on midday leaf water potential (ψ_leaf_) and hydraulic conductance of soil and plants (Sperry and Love 2015, Sperry et al. 2017, Carminati and Javaux 2020, Joshi et al. 2022); and (ii) studies emphasizing the role of water storage capacity (i.e., the total amount of internally stored water that can be mobilized to support transpiration; distinct from hydraulic capacitance, which describes the change in water storage per unit change in water potential), nighttime stem rehydration and predawn water potential (ψ_leaf,pd_) as the key state variables for predicting stomatal closure (Zweifel et al. 2021*b*, Peters et al. 2023, 2025, Potkay and Feng 2023). The aim of our study is to provide a conceptual model linking the midday and the night view of stomatal regulation of water use.

We begin with the midday approach. Several successful models of stomatal response to soil moisture and VPD are based on the water supply–demand framework (Sperry and Love 2015, Sperry et al. 2017, Carminati and Javaux 2020, Joshi et al. 2022, Carminati et al. 2026). These models describe water flow from the soil to the leaf, driven by gradients in water potential, and controlled by the hydraulic conductance of key plant elements (soil, root, stem and leaves). These models assume that plants optimize carbon assimilation over a water cost, which has been associated with the midday leaf water potential (Sperry et al. 2017, Joshi et al. 2022). According to these models, plants minimize the risk of excessively negative leaf water potential (e.g., to prevent embolism formation in the xylem) by downregulating stomatal conductance when the atmospheric water demand exceeds the plant’s capacity to transport water and/or the soil’s ability to supply water to the roots.

However, these models neglect water storage capacity, which can buffer transient diurnal imbalances between water supply and transpirational demand and is replenished during periods of low transpiration (e.g., at night; Čermák et al. 1976). Specifically, water dynamics in trees reflect the interaction between root water uptake and atmospheric demand, resulting in alternating phases of storage depletion and rehydration (Steppe et al. 2006, Brum et al. 2026). Importantly, these processes can be observed through variations in stem diameter across both daily and seasonal timescales (Hinckley and Bruckerhoff 1975, Zweifel et al. 2001). While stem diameter changes in this study are primarily interpreted as reflecting fluctuations in water storage, they do not exclusively represent reversible water storage dynamics (Brum et al. 2026). Notably, the physiologically relevant variable is turgor pressure (Bartlett et al. 2012). Turgor is essential for irreversible cell expansion and thus growth (Lockhart 1965, Steppe et al. 2006). Trees that recover faster from daytime dehydration and turgor loss have greater potential for nighttime growth (Zweifel et al. 2021b). Consistently, temperate tree species reduce their stomatal conductance in order to maintain nighttime turgor, which supports cell expansion (Peters et al. 2023, Potkay and Feng 2023). Along this line, it was shown that stomatal conductance during the day is better predicted by ψ_leaf,pd_ than ψ_leaf,md_ (Peters et al. 2025). These results highlight the importance of nighttime recovery processes for plant water use.

While the effects of soil drying on daytime plant water transport are well established (Sperry and Love 2015, Carminati and Javaux 2020), its consequences for nighttime recovery remain poorly understood. This knowledge gap arises because existing hydraulic supply–demand models neglect internal water storage and its depletion during the day and replenishment at night. To address this limitation, we incorporate mass conservation into an established supply–demand framework to develop a conceptual model that links daytime water depletion with nighttime recovery, providing a hydraulic basis for understanding how soil drying constrains both processes. The approach is grounded in physical principles and provides an upper constraint on plant water use while recognizing that plant adjustments also contribute to shaping hydraulic responses.

We describe the mass balance of a tree by equating changes in internal water storage to the net difference between water supply and transpiration. The water mass balance is given by:


(1) \begin{equation*} {\int}_{\mathrm{t}1}^{\mathrm{t}2}S(t)\ \mathrm{dt}-{\int}_{\mathrm{t}1}^{\mathrm{t}2}D(t)\ \mathrm{dt}=\Delta V={\int}_{\mathrm{\psi} 1\left(\mathrm{t}1\right)}^{\mathrm{\psi} 2\left(\mathrm{t}2\right)}C\left(\mathrm{\psi} \right)d\mathrm{\psi} \end{equation*}

where *S*(*t*) is the supply rate supplying water from soil to the plant (m^3^ s^−1^), *D*(*t*) is the transpiration rate (m^3^ s^−1^) and ∆*V* is the net change in internal water storage. The function *C*(ψ) denotes the hydraulic capacitance of the system, defined as $C\left(\mathrm{\psi} \right)= dV/\mathrm{d}\mathrm{\psi}$, where *V* is water storage and ψ is plant water potential. The terms ψ_1_(*t*_1_) and ψ_2_(*t*_2_) denote plant water potential at the beginning (*t*_1_) and end (*t*_2_) of the diurnal cycle, respectively.

Using the mass-balance idea, daytime depletion Δ*V*_day_ and nighttime rehydration Δ*V*_night_ can be expressed as functions of water supply and demand, thereby linking internal water storage to hydraulic supply–demand models. Δ*V*_day_ begins at *t*_onset_, when transpiration exceeds water supply (*D>S*), resulting in a net depletion of stored water:


(2) \begin{equation*} {\int}_{t_{onset}}^{t_{switch}}\left(S(t)-D(t)\right)\mathrm{dt}=\Delta{V}_{\mathrm{day}}\kern0.5em \end{equation*}

where *t*_switch_ marks the phase-shift when water supply exceeds transpiration (*S* > *D*). Δ*V*_night_ spans from *t*_switch_ until predawn of the following day (*t*_predawn_) and represents the recovery of water storage:


(3) \begin{align*} {\int}_{t_{\mathrm{switch}}}^{t_{predawn}}\left(S(t)-D(t)\right)\mathrm{dt}=\Delta{V}_{\mathrm{night}} \end{align*}

By combining these storage terms with functions describing the transpiration rate and the water supply rate (see Materials and  methods), we investigate how Δ*V*_day_ and Δ*V*_night_ evolve during progressive soil drying. The resulting conceptual model is shown in Figure 1. Under wet conditions (Figure 1a), supply exceeds or matches demand, and no substantial storage buffering is required. With moderate drying (Figure 1b), Δ*V*_day_ increases, which, in turn, increases Δ*V*_night_. However, with further soil drying (Figure 1c), Δ*V*_night_ can no longer increase because declining supply rates constrain nighttime rehydration. Consequently, with progressive soil drying, Δ*V*_day_ could theoretically continue to increase (provided stomata remain open), whereas Δ*V*_night_ reaches a maximum and subsequently declines due to soil hydraulic limitation (Figure 1d). We define the potential at which Δ*V*_night_ reaches its maximum as critical soil water potential (ψ_soil,crit_). Beyond this threshold, maintaining high transpiration rate becomes increasingly risky. We therefore hypothesize that the onset of stomatal regulation is concomitant with the hydraulic threshold ψ_soil,crit_, defined by the maximum in nighttime rehydration.

**Figure 1:**
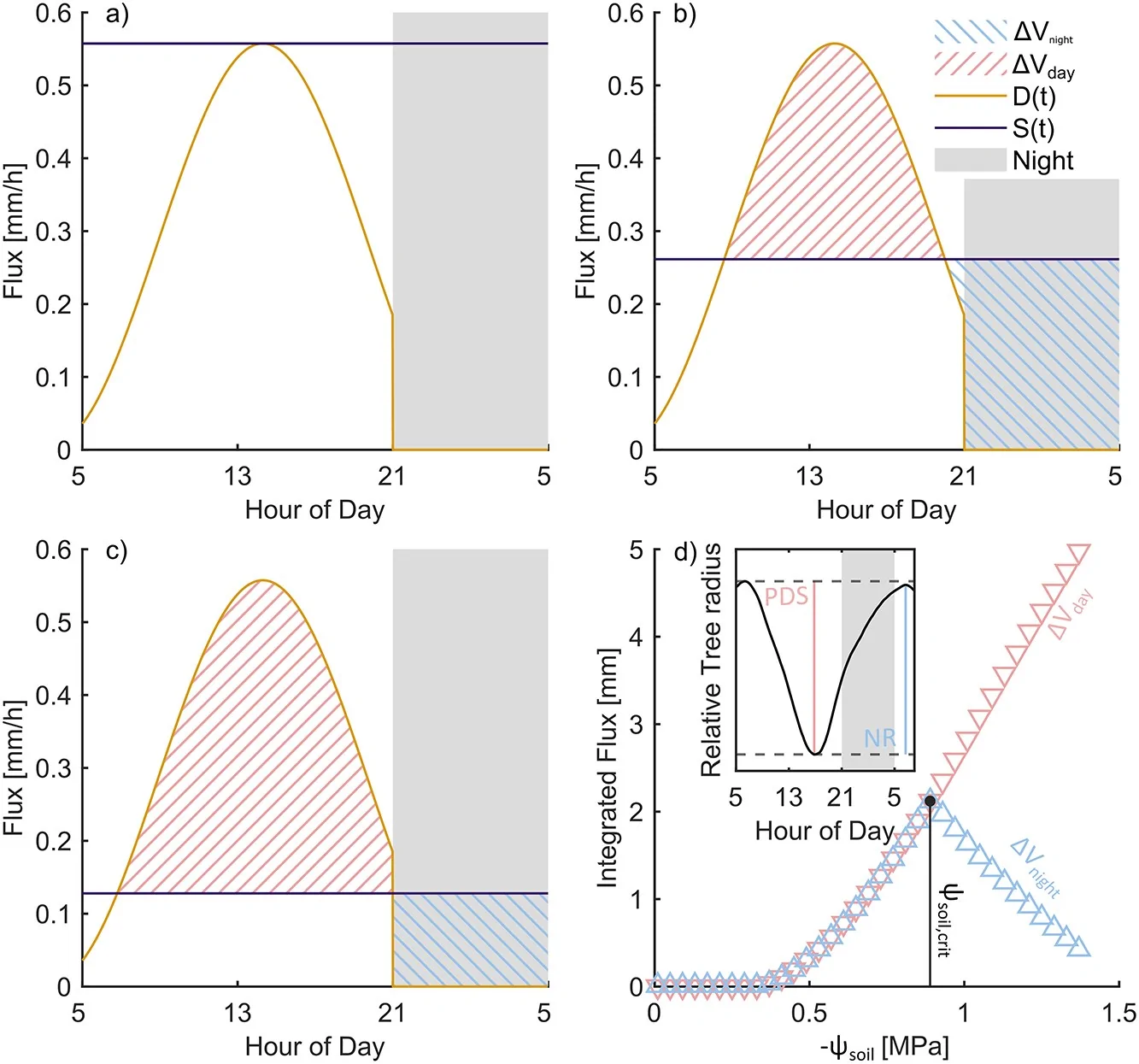
Conceptual model of diurnal water storage dynamics under soil drying. (a–c) Progressive soil drying reduces water supply (*S*(*t*)) due to declining soil hydraulic conductance, represented by the declining position of the horizontal line across panels, while atmospheric demand (*D*(*t*)) follows a seasonally constant diurnal cycle (grey shading indicates nighttime). Right-leaninghatched areas represent daytime depletion (Δ*V*_day_; *D*(*t*) > *S*(*t*)), and left-leaninghatched areas represent nighttime recovery (Δ*V*_night_; *S*(*t*) > *D*(*t*)). With progressing soil drying, (a) supply meets or exceeds demand, (b) depletion and recovery increase but remain balanced, and (c) depletion increases while recovery declines. (d) Integrated daytime depletion (Δ*V*_day_) and nighttime recovery (Δ*V*_night_) as a function of soil water potential (ψ_soil_), showing increasing depletion and declining recovery. The threshold ψ_soil,crit_ marks the onset of imbalance where Δ*V*_day_ > Δ*V*_night_. The inset illustrates the field-based metrics peak diurnal shrinkage (PDS) and nighttime recovery (NR) derived from stem radius variations.

To test this hypothesis in the field, we link Δ*V*_day_ and Δ*V*_night_ to observable quantities. We define peak diurnal shrinkage (PDS; often referred to as maximum daily shrinkage, e.g., Ortuño et al. 2010, Fernández and Cuevas 2010, Dietrich et al. 2018) as the realized outcome of ΔV_day_, derived from the difference between predawn and midday stem radius. Similarly, nighttime rehydration (NR), defined as the increase in stem radius from midday minimum to the subsequent predawn, represents the realized Δ*V*_night_. Both PDS and NR represent integrated responses to the underlying balance between water supply and demand, without explicitly resolving them. In the field, based on the conceptual model, we expect increasing PDS and NR values with progressive drying, followed by a sharp decline at potentials concomitant with the onset of stomatal regulation.

## Materials and methods

### Study sites

Field studies were conducted in Valais, Switzerland, at two forest sites that are part of two research platforms (Forest Soil Database and TreeNet; Zweifel et al. 2021*a*) managed by the Swiss Federal Institute for Forest, Snow, and Landscape Research (WSL). These sites are covered by mixed downy oak (*Quercus pubescens Willd.*) forests that have remained unmanaged for decades. Table 1 provides an overview of their key climatic characteristics. Both sites experience a hot and dry climate during the growing season.

**Table 1:** Topography and climate at the study sites in Valais, Switzerland. Mean annual temperature (MAT) and mean annual precipitation (MAP) are from Walthert et al. (2024).

**Site**	**Lat**	**Long**	**Altitude**	**MAT**	**MAP**
	**(N°)**	**(E°)**	**(m.a.s.l.)**	**(°C)**	**(mm)**
Saillon	46.17	7.17	790	9.2	783
Chippis	46.30	7.58	632	9.8	639

### Sap flow measurements

In March 2023, at each site, two trees were instrumented with one sap flow sensor (UGT SFM-4, Müncheberg, Germany) installed at breast height. For installation, the bark around the sensor was carefully thinned to a remaining thickness of 5 mm. For the three needles of the sensor (two needles with a pair of temperature sensors and a heater probe in the middle), three holes were drilled, and the needles with temperature sensors at 10 and 20 mm were inserted into the sapwood. Data were recorded at 15-min intervals, and sap flux density of inner and outer sensor position was calculated following the method described in Forster (2020). In short, the sap flow of the entire stem was estimated by scaling the inner and outer sap flux densities according to sapwood area. Sapwood thickness was taken as 49 mm based on literature for this species (Poyatos et al. 2007), with the inner and outer sapwood layers separated at 15 mm below the cambium. Although the inner sapwood area was larger, it contributed less to total sap flow due to lower measured velocities. Final sap flow was calculated based on:


(4) \begin{equation*} SF={J}_{inner}\cdotp Sapwoo{d}_{inner}+{J}_{outer}\cdotp Sapwoo{d}_{outer} \end{equation*}

where SF is the sap flow (cm_3_ h_−1_), *J*_inner_ and *J*_outer_ is the inner and outer sap flux density (cm^3^ cm^−2^ h^−1^) and Sapwood_inner_ (cm^2^) and Sapwood_outer_ (cm^2^) are the respective sapwood areas (Marshall 1958). Parameters used for these calculations are shown in Table 2. To correct for potential zero-flow offsets, data from 22 March to 5 April (when sap flow was minimal) were used to correct baseline offset (Clearwater et al. 2009). To then derive daily peak sap flow, we calculated the 95th percentile of 15-min sap flow measurements for each day. This approach reduces sensitivity to outliers while preserving the peak. Furthermore, sap flow was normalized by potential evapotranspiration (PET) to assess stomatal regulation. This approach reflects the fact that transpiration (sap flow) is governed by the combined effects of stomatal conductance and atmospheric demand (e.g., Jarvis et al. 1976). Specifically, we divided by the daily sap flow with the daily potential evapotranspiration (PET; MeteoSwiss), which integrates radiation, temperature, humidity and wind, and serves as a proxy for atmospheric demand.

**Table 2:** Tree characteristics, at breast height, relevant for calculating sapflow for the four investigated Quercus pubescens trees. Trees A and B were located on the coarse site, whereas Trees C and D were located on the fine site (Table 4).

**Site and tree**	Circumference (cm)	Bark thickness (cm)	Inner sapwood area (cm^2^)	Outer sapwood area (cm^2^)
**Chippis Tree A**	63	1.3	118	75
**Chippis Tree B**	51	1.3	77	57
**Saillon Tree C**	76	2.4	139	84
**Saillon Tree D**	67	2.1	115	74

### Soil matric potential measurements

Soil matric potential (ψ_soil_) was measured using two or three MPS-2 dielectric sensors (Decagon Devices, Pullman, WA, USA) installed in the soil profile wall at defined depth layers (Table 3) in the year 2014. These sensors determine dielectric permittivity of two porous ceramic discs, which varies with their water content. Matric potential was derived from the known relationship between water content and matric potential of the discs. Data were logged hourly using Decagon EM-50 data loggers. Sensor readings were corrected following established protocols (Walthert and Schleppi 2018, Walthert et al. 2023). As all four soil depths exhibited similar drying dynamics, we used the arithmetic mean across depths as a representative estimate of ψ_soil_ for comparison with plant physiological parameters.

**Table 3:** Overview of soil sensors depths.

**Site**	**Sensors 1–2**	**Sensors 3–5**	**Sensors 6–7**	**Sensors 8–10**
**Saillon**	20 cm	80 cm	140 cm	200 cm
**Chippis**	20 cm	80 cm	140 cm	190 cm

### Tree water storage dynamics derived from dendrometer measurements

To measure stem radius changes, we used automated high-precision point dendrometers (ZN12-T-WP, Natkon, Oetwil am See, Switzerland). At each site, two trees were instrumented with one dendrometer each, installed at breast height. The processing followed established methods (Knüsel et al. 2021, Luković et al. 2022). To quantify diurnal storage dynamics, we derived PDS and nighttime rehydration (NR) from stem radius (SR) measurements (see Figure 1). Peak diurnal shrinkage was calculated as the difference between maximum stem radius in the morning (SR_morning_) and the subsequent minimum during midday (SR_midday_):


(5) \begin{equation*} \mathrm{PDS}=\mathrm{S}{\mathrm{R}}_{\mathrm{morning}}-\mathrm{S}{\mathrm{R}}_{\mathrm{midday}} \end{equation*}

Nighttime rehydration was calculated as the minimum of PDS and the increase in stem radius from the midday minimum (SR_midday_) to the following morning maximum (SR_next morning_): 


(6) \begin{equation*} \mathrm{NR}=\min \left( PDS,S{R}_{\mathrm{next}\ \mathrm{morning}}-S{R}_{\mathrm{midday}}\right) \end{equation*}

Here, SR_morning_ and SR_next morning_ are defined as the maximum stem radius recorded between 00:00 and 12:00 h on the respective days. The use of the minimum function excludes the contribution of irreversible radial growth, thereby isolating the reversible component of stem radius variation.

### Nighttime equilibrium

To assess whether the tree equilibrates with the soil overnight, continuous measurements of leaf water potential were required. Following recent work, we fitted an empirical equation (see Table 4 for the corresponding parameter values) to predict ψ_leaf_ based on tree water deficit (TWD) (Dietrich et al. 2018). Tree water deficit was calculated based on the zero-growth concept (Zweifel et al. 2016). For simplicity, we used the following fitting approach for the calibration data:


(7) \begin{equation*} {\mathrm{\psi}}_{leaf}=a\cdotp \sqrt{TWD}+b\cdotp TWD \end{equation*}

The fit explained a large proportion of the observed variation in leaf water potential (*R*^2^ = 0.76–0.96). To calibrate the equation, we used leaf water potential (ψ_leaf_) measurements collected following Walthert et al. (2024), where data for the Chippis site have already been published. In brief, measurements were taken from leaves of the study trees using a Scholander-type pressure chamber (Model 1000, PMS Instruments, Albany, OR, USA) during the vegetation period on 14 rainless days between 2017 and 2020. For further details, we refer to Walthert et al. (2024).

**Table 4:** Calibrated parameter values for the model predicting (ψ_leaf_) and the goodness of fit, evaluated using the adjusted *R*^2^ (*R*^2^adj). Leaf water potential minimum (LWP_min_) and maximum (LWP_max_) values, illustrating the full range observed for each tree.

** Tree**	**a**	**b**	***R*** ^**2**^ _**adj**_	**LWP** _**max**_ **(MPa)**	**LWP** _**min**_ **(MPa)**
**Tree A**	0.0019	0.0099	0.88	−0.27	−4.13
**Tree B**	0.0181	0.0077	0.96	−0.32	−4.63
**Tree C**	0.098	0.0019	0.86	−0.27	−3.53
**Tree D**	0.087	0.0026	0.76	−0.32	−3.97

### Data selection criteria

To minimize the influence of phenological changes (e.g., leaf unfolding and senescence) on transpiration measurements, we restricted the analysis to the summer months (June–August), corresponding to full canopy development with relatively stable leaf area and consistently high potential transpiration rates. To ensure consistency with the conditions under which leaf water potential was calibrated (Table 4), only days with potential evapotranspiration (PET) ≥ 3 mm day^−1^ and precipitation ≤1 mm were included. This restricts the analysis to remain within the range of environmental conditions represented in the calibration data.

### Statistical analysis

To identify threshold behaviour in hydraulic variables, segmented regression analysis was applied using the R package ‘segmented’ (Muggeo 2008; Version 2.1-4). For each tree and response variable, linear models were first fitted to describe the relationship between the response (e.g., PDS, NR, normalized sap flow, ψ_leaf,pd_ and the predictor ψ_soil_. These models were then extended to segmented regressions to estimate breakpoints ψ_soil,crit_ representing shifts in system behaviour. Breakpoint estimates and their 95% confidence intervals were calculated using the ‘segmented’ package based on the fitted model. Confidence intervals were derived using the default option implemented in the package. All analyses were performed in R (version 4.4.2; R Core Team, 2024).

### Conceptual model

To estimate daytime and nighttime storage dynamics (Δ*V*_day_ and Δ*V*_night_), the model requires both a soil water supply *S*(*t*) and an atmospheric demand *D*(*t*). For the supply term *S*(*t*), we used the model of Carminati and Javaux (2020), with parameter values listed in Table 5. This model describes water flow from the bulk soil to the leaf as a function of ψ_soil_ and ψ_leaf_ through a series of hydraulic resistances. For conceptual simplicity, we assumed a constant ψ_leaf_ of −1.5 MPa and a stepwise decreasing ψ_soil_ from −0.01 to −1.4 MPa. As the conceptual model purposely neglects stomatal regulation, transpiration was assumed to follow atmospheric demand. Accordingly, *D*(*t*) was derived from potential evapotranspiration data obtained from a nearby meteorological station (MeteoSwiss). To represent a typical hot summer day, hourly data from the 10 hottest days of a given year were used to fit a sinusoidal function: 


(8) \begin{align*} AtmD=a\cdotp \sin \left(b\cdotp time+c\right)+d\kern0.5em \end{align*}

where AtmD is the atmospheric demand [mm/d]; a, b, c and d are fitting coefficients; and time is the hour of the day. Within the night (21:00 to 05:00 h) the AtmD was set to zero. This simplification was chosen to reduce model complexity. Although atmospheric demand is not strictly zero during the night, we assume that the simplification mainly affects the magnitude of the simulated response rather than the underlying processes.

**Table 5:** Parameters used in the Carminati and Javaux (2020) model.

**Parameter**	**Value**
Soil air-entry value *h*_0_ (cm)	−3.58
Soil pore size distribution parameter λ (−)	0.211
Soil saturated hydraulic conductivity k0 (cm/s)	2.694·10^−4^
Soil tortuosity parameter τ (−)	2.633
Root resistance *R*_root_ (hPa cm^−3^∙s)	7·10^−4^
Root mean radius *r*_0_ (cm)	0.1
Radius of bulk soil *r*_2_ (cm)	1
Root length L (cm)	1·10^8^
P50 h_0x_ (cm)	−24,500
Xylem tortuosity τ_x_ (−)	5

## Software

MATLAB (The Mathsworks Inc. 2021) was used to conduct the data analysis and to create all figures.

## Results

We investigated how peak diurnal shrinkage (PDS) and nighttime rehydration (NR) change with decreasing ψ_soil_ and whether these changes are reflected in stomatal regulation. Figure 2 shows four trees distributed across two sites (Trees A and B; Trees C and D), relating soil water potential to PDS, NR, normalized sapflow and ψ_leaf,pd_. Sap flow measurements were unavailable for Tree A.

**Figure 2:**
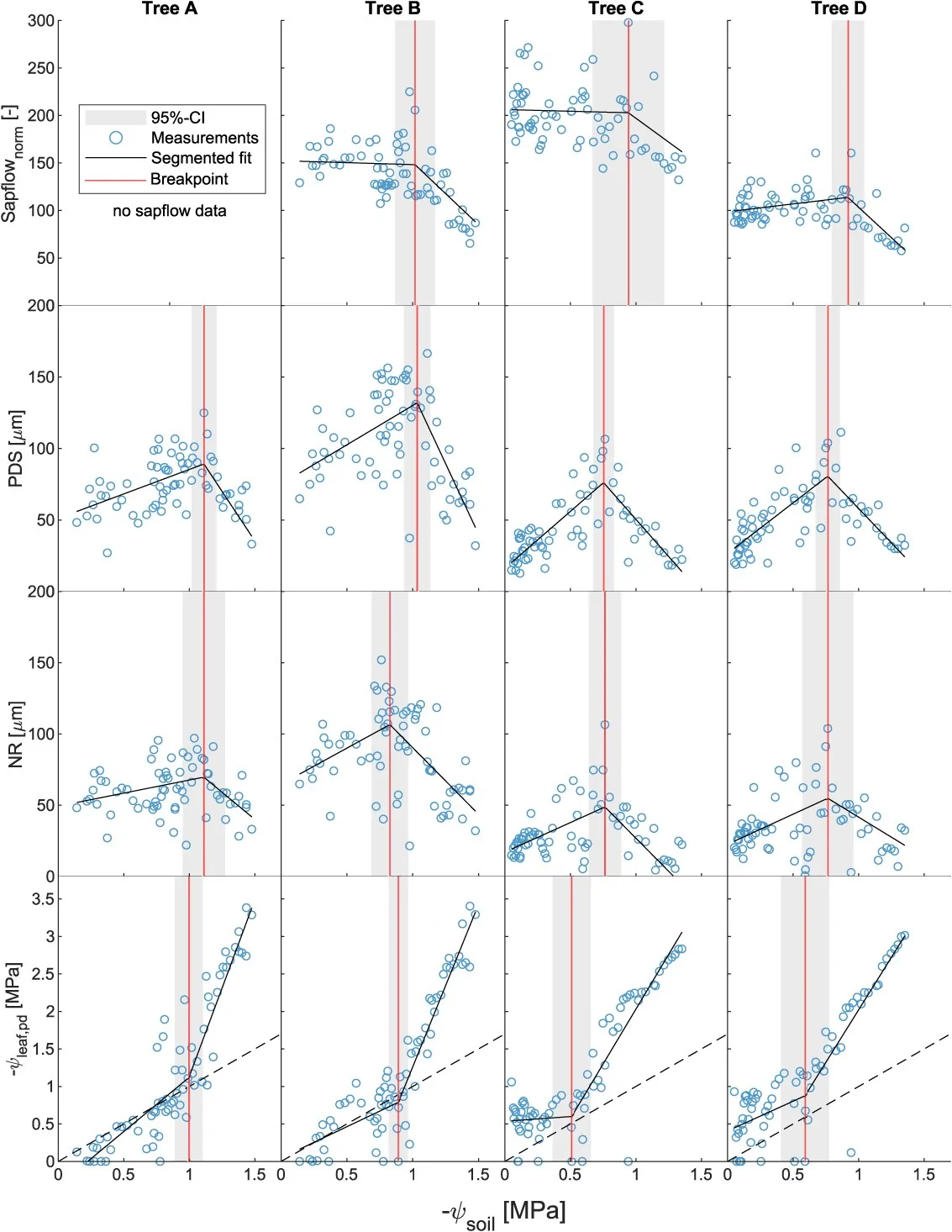
Estimated ψ_soil,crit_ for four trees based on four key parameters. Relationships between soil water potential (ψ_soil_; depth-averaged; see Materials and methods section) and key hydraulic variables on a daily scale for four *Quercus pubescens* trees (a–d) across two sites. Rows represent (from top to bottom): normalized sap flow (proxy for transpiration rate and stomatal downregulation), peak diurnal shrinkage (PDS), nighttime stem radius recovery (NR) and predawn leaf water potential (ψ_leaf,pd_). The dashed line in the bottom row represents the expected predawn condition if the plant has fully rehydrated overnight relative to soil water potential (ψ_leaf,pd_ = ψ_soil_). Breakpoints consistently emerge across variables, indicating that trees regulate stomatal conductance to maintain a balance between daytime depletion (PDS) and nighttime recovery (NR), with ψ_soil,crit_ marking the transition beyond which this balance can no longer be maintained.

Across all trees, the measured variables exhibited characteristic responses to soil drying (Figure 2). The PDS and NR initially increased with decreasing ψ_soil_, reached a maximum and subsequently declined under drier conditions. In contrast, normalized sap flow remained relatively constant under wet conditions before declining with further soil drying. Likewise, ψ_leaf,pd_ initially followed ψ_soil_, but transitioned to a steeper decline under drier conditions. These response patterns were quantified using segmented regression (see Materials and methods). Breakpoint estimates were remarkably consistent among variables within each tree and exhibited overlapping 95% confidence intervals (Table 6). Within sites, breakpoint estimates were also similar, averaging 1.1 and 0.9 MPa for Trees A and B, respectively, and ~0.7 MPa for Trees C and D. Consequently, variation among sites exceeded variation among trees within a site. Based on the fitted regressions, peak PDS values ranged from ~70 to 130 μm, whereas peak NR values ranged from 45 to 105 μm. In all trees, maximum nighttime rehydration remained lower than maximum daytime depletion.

**Table 6:** Mean ± standard deviation of mean absolute ψ_soil_ for physiological breakpoints of four trees (A--D), including sapflow (proxy for transpiration or stomatal conductance), daytime deficit, night recovery, and ψ_leaf,pd_. All units are in MPa.

** Breakpoints**	**Tree A**	**Tree B**	**Tree C**	**Tree D**
Sap flow	NaN	1.02 ± 0.15	0.94 ± 0.27	0.92 ± 0.12
Daytime deficit	1.11 ± 0.10	1.03 ± 0.10	0.75 ± 0.08	0.76 ± 0.09
Night recovery	1.11 ± 016	0.83 ± 0.14	0.76 ± 0.12	0.76 ± 0.20
ψ_leaf,pd_	1.00 ± 0.11	0.89 ± 0.07	0.51 ± 0.15	0.59 ± 0.18
Mean	1.07	0.92	0.68	0.71

We further examined the relationship between ψ_leaf,pd_ and ψ_soil_ to assess whether equilibrium between plant and soil persists under progressive drying (Figure 2, bottom panels). Under wet conditions, ψ_leaf,pd_ broadly follows the expected 1:1 relationship with ψ_soil_ (dashed black line). However, beyond the breakpoint, ψ_leaf,pd_ deviates systematically from this equilibrium across all trees. We interpret this breakpoint as the point when nighttime soil water flow can no longer refill the trees up to the point that the bulk soil water potential would allow. This breakpoint occurs at soil water potentials comparable to those identified for PDS, NR and normalized sapflow (Table 6), supporting our interpretation that declines in nighttime rehydration are rate and soil limited. Together, these observations indicate that changes in storage dynamics, transpiration and predawn equilibration are concomitant and occur within a similar range of a critical soil water potential (ψ_soil,crit_).

## Discussion and conclusions

### ψ_soil,crit_ as critical threshold for onset of stomatal regulation

We hypothesized that the onset of stomatal regulation is concomitant with the hydraulic threshold defined by the maximum in nighttime rehydration (ψ_soil,crit_). The field results are consistent with this expectation: Figure 2 shows that the onset of reduction in normalized sapflow (reflecting stomatal regulation) is concomitant with the decline in both PDS and NR. We interpret ψ_soil,crit_ as an upper operating boundary, rather than a strict failure point, as trees may reduce transpiration earlier. Similarly, some species might also move beyond this operating boundary, but this would be a risky strategy, as these trees would face accelerating dehydration.

The value of ψ_soil,crit_ is determined by factors that influence the balance between water supply and atmospheric demand. Increased atmospheric demand enhances daytime shrinkage, while reduced soil water availability and declining soil–root hydraulic conductance limit water supply. In addition, tree physiological traits influence both depletion and recovery rates, including adjustments in hydraulic conductance, leaf area, turgor loss point and root properties observed under drought conditions (Limousin et al. 2009, Bartlett et al. 2012, Brunner et al. 2015, Moreno et al. 2021). These responses suggest that the hydraulic functioning of the system reflects not only current environmental conditions but also prior exposure to drought. As a result, ψ_soil,crit_ should be understood as a dynamic operating boundary emerging from the interaction between atmospheric demand, soil properties and plant hydraulic traits. While this threshold theoretically might vary across ecosystems, for temperate tree species it may converge toward similar values (Peters et al. 2025), likely reflecting constraints imposed by declining soil hydraulic conductivity on water extraction as soils dry (Carminati et al. 2026).

The dynamic operating boundary, in combination with the conceptual model, provides a mechanistic basis for the well-established role of turgor in controlling growth (Lockhart 1965, Steppe et al. 2006). Within this framework, the timing of nighttime growth and the reduction in growth under high atmospheric demand (Zweifel et al. 2021b) can be understood as consequences of the diurnal water balance. Increased daytime depletion elevates the demand for nighttime recovery, which may be limited by hydraulic constraints, thereby delaying the restoration of turgor and constraining growth.

ψ_soil,crit_, together with Δ*V*_day_ and Δ*V*_night_, provides a unifying framework that links daytime demand–supply dynamics with nighttime recovery processes. In that sense, the model reconciles perspectives focusing on hydraulic limitation during the day (Sperry and Love 2015, Sperry et al. 2017, Carminati and Javaux 2020, Joshi et al. 2022) with those emphasizing nighttime recovery and growth (Zweifel et al. 2021b, Peters et al. 2023, 2025, Potkay and Feng 2023).

### Nighttime equilibrium

Under wet conditions (ψ_soil_ > ψ_soil,crit_), ψ_leaf,pd_ closely approaches ψ_soil,pd_, indicating effective equilibration between plant and soil over the night (Figure 2, last row). Importantly, this balance does not imply complete rehydration, as progressive soil drying lowers ψ_soil_ and ψ_leaf,pd_ thereby shifting the plant to an increasingly drier state. As soils dry, NR initially increases, suggesting that plants can enhance nighttime rehydration in response to greater daytime depletion. However, upon reaching ψ_soil,crit_, this trend reverses. Recovery no longer increases despite continued drying, and the plant fails to equilibrate with the soil, despite PDS declining. Below this threshold (ψ_soil_ < ψ_soil,crit_), plant water potential declines more rapidly than ψ_soil_, indicating that nighttime rehydration becomes insufficient to offset daytime losses. Predawn disequilibrium between plant and soil water potentials is well documented and often attributed to nighttime transpiration (e.g., Donovan et al. 2001, 2003, Bucci et al. 2004). While such losses can contribute to incomplete recovery, our analysis suggests a complementary mechanism in which the rate of nighttime rehydration itself becomes limiting, thereby constraining NR. In this view, predawn disequilibrium reflects both ongoing water losses and limitations in nighttime rehydration. Alternatively, the observed decline in NR and emergence of predawn disequilibrium may also be interpreted within the broader context of stem rehydration dynamics and hydraulic strategies proposed for temperate tree species during drought (Peters et al. 2023, 2025, Brum et al. 2026).

## Data Availability

The data are available at https://doi.org/10.6084/m9.figshare.31018087. The repository includes: (i) Measurements of stem radius (10 min resolution), inner and outer sap flux densities for each tree (15 min resolution), and soil water potential (ψ_soil_; 60 min resolution) from June 2023 to August 2023; (ii) processed data used to generate Figure 2; (iii) concomitant measurements of ψ_leaf_ and TWD from 14 days (2017–20). All data required to reproduce the analyses and figures presented in this study are provided.
